# The Rule of Three

**Published:** 1917-12

**Authors:** 


### The Rule of Three.

2,000,000 : 20,000 :: 4,000,000 : 12,000. It is not correct mathematically but it is all right according to various authoritative statements. If it will take at least 20,000 surgeons to care for an American Army of 2,000,000, how is it that we are assured that the British (meaning British Isles)

Army numbers 4,000,000 and that the total number of English Army surgeons is only 12,000?

